# Lack of Protection Against Ebola Virus from Chloroquine in Mice and Hamsters

**DOI:** 10.3201/eid2106.150176

**Published:** 2015-06

**Authors:** Darryl Falzarano, David Safronetz, Joseph Prescott, Andrea Marzi, Friederike Feldmann, Heinz Feldmann

**Affiliations:** National Institute of Allergy and Infectious Diseases, Hamilton, Montana, USA

**Keywords:** Ebola virus infection, antiviral drug, animal model, chloroquine, treatment, viruses, antimicrobial drugs

## Abstract

The antimalarial drug chloroquine has been suggested as a treatment for Ebola virus infection. Chloroquine inhibited virus replication in vitro, but only at cytotoxic concentrations. In mouse and hamster models, treatment did not improve survival. Chloroquine is not a promising treatment for Ebola. Efforts should be directed toward other drug classes.

Chloroquine was first used as an antimalarial drug until widespread resistance in *Plasmodium falciparum* strains emerged. However, for >30 years this drug also has been recognized as having broad-spectrum antiviral properties (*1*), including activity against HIV-1 (*2*); the human coronaviruses, severe acute respiratory syndrome coronavirus (*3*) and OC43 (*4*); dengue virus (*5*); chikungunya virus (*6*); and influenza virus (*7*) in cell culture. Despite these data, chloroquine is not approved for use against any viral infections.

Previous in vitro data state a half maximal effective concentration (EC_50_) and EC_90_ of 16 and 25 mol/L for chloroquine against Ebola virus (EBOV), respectively (*8*). Twice daily dosing at 90 mg/kg intraperitoneally rapidly achieved a steady-state concentration of 2.5 μg/mL in the blood of mice. This dosing regimen resulted in survival of 85% of mice after infection with mouse-adapted (MA) EBOV (*8*). These data have led to the suggestion that chloroquine and its derivatives be used in persons with EBOV infection because this drug is approved for use in humans, has an extensive safety profile, and is inexpensive (*1*,*9*). To determine whether protection would extend to the EBOV hamster model, during 2013–2014 we investigated chloroquine treatment in this model and attempted to repeat previous in vitro findings and findings in the mouse model.

## The Study

Vero E6 cells were infected with 100 focus-forming units of EBOV expressing enhanced green fluorescent protein. After a 1-h incubation, the inoculum was removed and replaced with media (Dulbecco’s modified Eagle's medium with 2% fetal bovine serum, Penn/Strep, L-glutamine) containing chloroquine (Sigma, St. Louis, MO, USA). The supernatant was collected on days 1, 3, 5, 7, and 9 after infection and media replaced with fresh drug. Viral RNA was extracted from the supernatant and quantified by real-time quantitative reverse transcription PCR as previously described (*10*). Concurrently, cell viability was assayed by using Cell Titer96 Aqueous One Solution (Promega, Madison, WI, USA) according to the manufacturer’s instructions. EC was determined by using Prism6 (GraphPad Software, San Diego, CA, USA).

**Figure 1:**
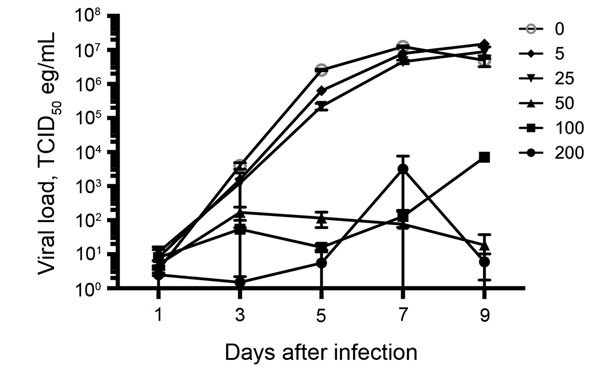
Viral loads from supernatants derived from Vero cells infected with Ebola virus expressing enhanced green fluorescent protein and treated with chloroquine at the indicated concentrations (0, 5, 25, 50, 100, and 200 μg/mL). TCID_50_, 50% tissue culture infectious dose.

When added 1 h after infection, chloroquine at 5 μg/mL and 25 μg/mL reduced the viral loads by 0.61 and 1.07 logs, respectively (peak reduction observed on day 5), without any significant cytotoxicity (Figure 1). Analysis of the data from day 5 resulted in an EC_50_ of 1.77 μg/mL and an EC_90_ of 23.34 μg/mL, concentrations that are comparable with previous data (*8*); however, reductions in viral loads at these concentrations at other time points were negligible. Although concentrations of >50 μg/mL of chloroquine reduced viral loads by 2–4 logs starting on day 3, this decrease was accompanied by a high level of cytotoxicity (>50%) that was evident both in the cytotoxicity assay and microscopically resulting in poor selectivity of chloroquine.

Six-week-old BALB/c mice or Syrian hamsters (both from Harlan, Indianapolis, IN, USA) were inoculated intraperitoneally with 100 50% lethal dose of MA EBOV. The mouse (*11*) and the hamster (*12*) are well-established disease models of EBOV infection. Treatment was initiated 1 h after inoculation. Treatment groups (mice and hamsters) received 90 mg/kg of chloroquine alone (intraperitoneally). Vehicle groups received the equivalent volume of sterile saline (intraperitoneally). Mock-infected animals received sterile tissue culture media in place of MA EBOV. An additional group of hamsters received 50 mg/kg of chloroquine (intraperitoneally every 24 h) in combination with 2.5 mg/kg doxycycline (gavage every 12 h) and 50 mg/kg azithromycin (intraperitoneally every 24 h). After inoculation, animals were monitored at least twice daily and euthanized by using a humane endpoint scoring criteria as approved by the Animal Care and Use Committee at Rocky Mountain Laboratories (Hamilton, MT, USA). Analysis of survival was performed in Prism6 (GraphPad).

**Figure 2:**
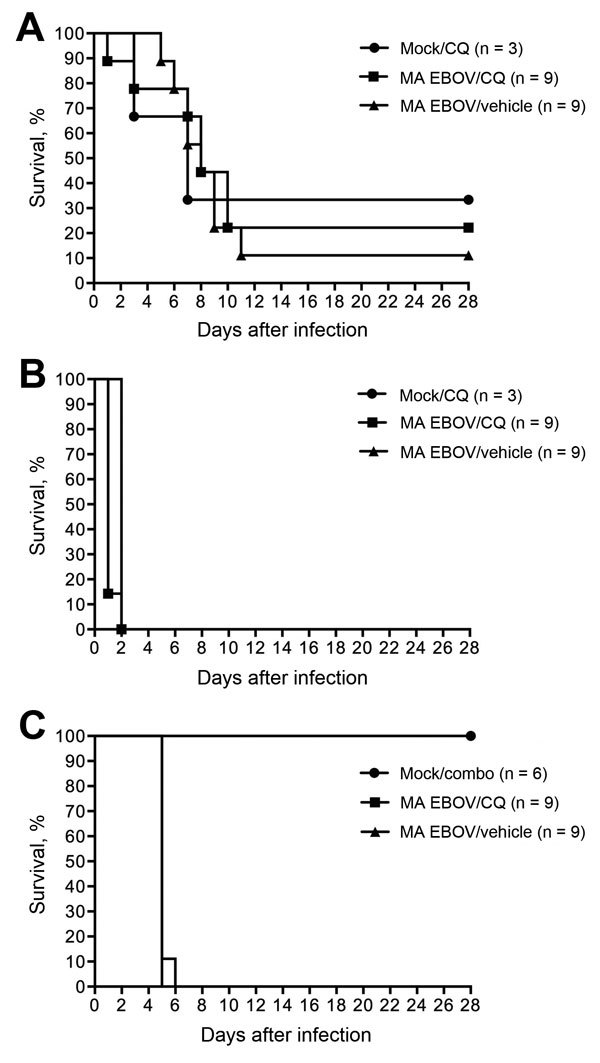
Survival of MA EBOV-inoculated mice (A) and hamsters (B) treated with CQ (90 mg/kg). C) Survival of MA EBOV–infected hamsters treated with a combination of CQ (50 mg/kg), doxycycline (2.5 mg/kg), and azithromycin (50 mg/kg). Combo, combination of chloroquine, doxycycline, and azithromycin; CQ, chloroquine; EBOV, Ebola virus; MA, mouse-adapted.

Two of 3 mock-challenged mice did not survive because of chloroquine (90 mg/kg) treatment alone (Figure 2, panel A). Only 2 of 9 mice infected with MA EBOV and treated with chloroquine survived, and 1 of 9 mice infected with MA EBOV and treated with vehicle survived. With median survival of 7, 8, and 8 d for mock-challenged/chloroquine-treated mice, MA EBOV–infected/chloroquine-treated mice, and MA EBOV–infected/vehicle-treated mice, respectively, treatment had no significant effect on survival. This dose, although previously stated as the maximum tolerated dose in mice (*8*), was not well tolerated by the animals in this study and clearly did not improve survival in animals challenged with MA EBOV.

When the same dose (90 mg/kg) of chloroquine was given to hamsters challenged with MA EBOV, the study had to be terminated on day 2 after treatment. Nearly all the treated animals, in both the MA EBOV and the mock-challenged groups, died of acute toxicity after administration of chloroquine intraperitoneally, typically within 30 min after treatment (Figure 2, panel B).

In a separate study, hamsters were treated with chloroquine (50 mg/kg) in combination with doxycycline (2.5 mg/kg) and azithromycin (50 mg/kg) to additionally provide broad-spectrum antimicrobial drug coverage. Reperfusion injury of the gut after EBOV disease, which would subsequently result in bacterial sepsis, has been suspected as a possible cause of death. Thus, broad-spectrum antimicrobial drugs were proposed to help in this regard. In this study, no toxicity was observed in the mock-challenged group as a result of the combination treatment. This finding suggests that hamsters tolerate this dose of chloroquine. However, treatment had no effect on survival; no combination-treated or vehicle-treated groups survived, and median survival times were comparable (Figure 2, panel C).

## Conclusions

Despite some activity of chloroquine against EBOV in vitro, we observed no benefit to its administration in the mouse and hamster models. In the mouse model, a dose of 90 mg/kg resulted in toxicity but did not alter survival; therefore, higher concentrations of chloroquine in the mouse would not be expected to be possible. In the hamsters, this dose was already lethal on its own. In the hamster model at a lower dose (50 mg/kg) combined with doxycycline and azithromycin—which together provide broad-spectrum antimicrobial coverage, in addition to doxycycline having a small antiviral effect against EBOV—survival did not change. Previous anecdotal reports of the incidental use of chloroquine in patients with filovirus infections also do not support any benefit from its use (*13*,*14*). Together, these data suggest that chloroquine is unlikely to provide any protection from EBOV infection in humans.

Given its in vitro activity against many different viruses and its longstanding use in humans, chloroquine has been put into multiple clinical trials. During dengue virus infection, viremia did not decrease (*15*), and chloroquine neither prevented influenza virus infection (*7*) nor improved outcome of chikungunya virus infection (*6*) despite promising in vitro activity against these viruses.

When taken together with previous findings for other less pathogenic viruses, the clinical use of chloroquine seems unlikely to provide any benefit for either prophylaxis or treatment of EBOV. Moreover, chloroquine has a small therapeutic window; dosing for treatment of acute malaria is ≈15 mg/kg, and lethality starts at 50 mg/kg. Thus, current preclinical data do not support the continued consideration of chloroquine for use against EBOV infections in humans.
